# Pathological ATX3 Expression Induces Cell Perturbations in *E. coli* as Revealed by Biochemical and Biophysical Investigations

**DOI:** 10.3390/ijms22020943

**Published:** 2021-01-19

**Authors:** Diletta Ami, Barbara Sciandrone, Paolo Mereghetti, Jacopo Falvo, Tiziano Catelani, Cristina Visentin, Paolo Tortora, Salvador Ventura, Antonino Natalello, Maria Elena Regonesi

**Affiliations:** 1 Department of Biotechnologies and Biosciences, University of Milano-Bicocca, 20126 Milan, Italy; diletta.ami@unimib.it (D.A.); barbara.sciandrone@unimib.it (B.S.); j.falvo@campus.unimib.it (J.F.); tiziano.catelani@unimib.it (T.C.); paolo.tortora@unimib.it (P.T.); 2 Milan Center of Neuroscience (NeuroMI), 20126 Milan, Italy; 3 Bioinformatics Consultant, 15061 Arquata Scrivia, Italy; paolo.mereghetti@gmail.com; 4 Department of Biosciences, Università degli Studi di Milano, 20133 Milan, Italy; Cristina.Visentin@unimi.it; 5 Institut de Biotecnologia i de Biomedicina and Departament de Bioquímica i Biologia Molecular, Universitat Autònoma de Barcelona, 08193 Barcelona, Spain; salvador.ventura@uab.cat

**Keywords:** ataxin-3 expression, amyloids, *Escherichia coli*, FTIR microspectroscopy, multivariate analysis, oligomer toxicity, protein aggregation

## Abstract

Amyloid aggregation of human ataxin-3 (ATX3) is responsible for spinocerebellar ataxia type 3, which belongs to the class of polyglutamine neurodegenerative disorders. It is widely accepted that the formation of toxic oligomeric species is primarily involved in the onset of the disease. For this reason, to understand the mechanisms underlying toxicity, we expressed both a physiological (ATX3-Q24) and a pathological ATX3 variant (ATX3-Q55) in a simplified cellular model, *Escherichia coli*. It has been observed that ATX3-Q55 expression induces a higher reduction of the cell growth compared to ATX3-Q24, due to the bacteriostatic effect of the toxic oligomeric species. Furthermore, the Fourier transform infrared microspectroscopy investigation, supported by multivariate analysis, made it possible to monitor protein aggregation and the induced cell perturbations in intact cells. In particular, it has been found that the toxic oligomeric species associated with the expression of ATX3-Q55 are responsible for the main spectral changes, ascribable mainly to the cell envelope modifications. A structural alteration of the membrane detected through electron microscopy analysis in the strain expressing the pathological form supports the spectroscopic results.

## 1. Introduction

Protein misfolding and subsequent aggregation in amyloid deposits are associated with more than 20 neurodegenerative diseases, commonly referred to as “protein misfolding diseases” or “conformational diseases” [1]. Examples of these pathologies are Alzheimer’s, Parkinson’s, prion, and polyglutamine (poly-Q) diseases. Distinct amyloidogenic polypeptides are implicated in the progression of these disorders. Protein aggregation into highly ordered β-sheet-rich supramolecular structures is a common feature of many polypeptide chains and a hallmark of the protein misfolding diseases [2,3], despite reduced sequence homology between amyloid-forming polypeptides.

The aggregation process from a native protein state into amyloid deposits implicates several steps. A general scheme provides a misfolded protein that assembles into low molecular weight oligomers, followed by the formation of larger aggregates, such as protofibrils and mature fibrils [4]. A different pathway to amyloid formation called “first-aggregation-and-then-misfolding” has also been identified, in which aggregation of globular proteins starts from conformational states close to native ones, with no need for transitions across the major energy barrier for unfolding [5]. One of the proteins that displays this behavior is the Josephin domain (JD) of ataxin-3 (ATX3) [6,7], the protein responsible for spinocerebellar ataxia of type 3 (SCA3), one of the ten known poly-Q disorders [8,9]. ATX3 consists of the N-terminal globular JD followed by an unstructured C-terminal region containing its poly-Q that triggers SCA3 when it exceeds a critical length of 50–55 consecutive residues [10,11]. This implies that the aggregation pathway consists of two steps: the first, only requiring the JD, gives rise to SDS-soluble protofibrils, and the second, solely accessible to variants carrying expanded poly-Qs, results in the formation of mature, SDS-insoluble fibrils [12,13,14]. Ruggeri and coauthors demonstrated the conformational transition of JD from native spheroidal oligomers, through misfolded oligomers, to the protofibrils [7]. In particular, the spheroidal oligomers contain JD with native-like secondary structure, while the conformational transition towards β-enriched structures occurs only later. This underlines the intermediate nature of the oligomers, some sharing the structural properties of native monomers (native-like), the others being quite similar to fibrils (fibril-like) [7].

The interest in oligomer polymorphism arises from the primary role of these species in cellular toxicity [15,16,17]. A leading theory on the molecular basis of amyloid toxicity argues that unstable early aggregates interact with cell membranes either non-specifically and/or at specific interaction sites, thus disturbing membrane ordered structure and/or impairing the activity of specific membrane proteins involved in signal transduction or ion transport, resulting in permeabilization or signaling deregulation [17,18,19]. Moreover, in SCA3, the species formed along the ATX3 self-assembly pathway seem to affect different cellular systems, including protein degradation pathways, gene transcription regulation, mitochondria and constituents implicated in the calcium homoeostasis [20,21,22,23]. It is challenging to discriminate if these effects are related to a toxic gain of function of the aggregated species or to a loss of the pleiotropic functions of ATX3. In this complex scenery, the employment of a simple transgenic system might be useful not only for better understanding the pathophysiological basis of the human disease, but also for the development of therapeutic approaches to treat this condition. *Escherichia coli* has been proved to be a simplified, but effective model system for the study of protein aggregation within a cellular environment [24,25]. An increasing number of published works support a similar relative aggregation propensity for amyloid proteins and protein mutants in humans and, when overexpressed, in bacteria. These results open the possibility of using *E. coli* for in-cell studies on protein aggregation and for high-throughput/ultrahigh-throughput functional screening of amyloid aggregation inhibitors [26,27]. Interestingly, different aggregated species of a chimeric protein containing a poly-Q tract have been detected during the heterologous expression in *E. coli*, allowing a better characterization of the complex aggregation process occurring in the cellular environment [25]. In a previous work, we used *E. coli* to reproduce the intracellular two-step aggregation pathway of the expanded ATX3 [28]. Noteworthy, in this model system the proteotoxicity of an expanded ATX3 carrying 55 Gln was found to correlate with the presence of soluble species and not with the amount of insoluble protein aggregates [28].

In this work, we took advantage of Fourier transform infrared (FTIR) microspectroscopy, supported by multivariate analysis, in combination with other biochemical and microscopical analyses, to study the mechanisms at the basis of ATX3 proteotoxicity. In particular, we investigated in situ the spectral changes that occur in intact *E. coli* cells expressing two ATX3 variants, the physiological Q24 and the pathological Q55, at two times of induction, i.e., when different protein aggregated species were formed. FTIR microspectroscopy is a non-invasive and label-free vibrational approach that gives a chemical fingerprint of the investigated sample, providing information on the content and structure of its main biomolecules (proteins, lipids, carbohydrates, and nucleic acids) [29]. Due to their complexity, the FTIR spectra of biological systems are the result of the overlapping absorptions of their main biomolecules. For this reason, to obtain the significant and non-redundant information contained in the spectra, the use of an appropriate multivariate analysis is necessary [30]. Here, we processed the FTIR data of intact *E. coli* cells expressing ATX3-Q24 and ATX3-Q55 by Partial Least Squares-Discriminant Analysis (PLS-DA), that enabled not only to evaluate the statistical significance of the observed spectral differences, but also to identify the spectral components responsible for the discrimination among the different analyzed classes, thus providing molecular-level information on the system under investigation.

Our biochemical and biophysical investigations allowed correlating the protein expression and aggregation with the induction of the cell perturbations. In particular, we showed that toxic oligomeric species of the pathological expanded ATX3 are responsible for the main spectral changes, ascribable mainly to the cell envelope modifications, in accordance with the severe membrane alterations observed through microscopic analysis.

## 2. Results

### 2.1. Toxic Effect of the Pathogenic Ataxin-3 Variant on E. coli Growth

**Figure 1:**
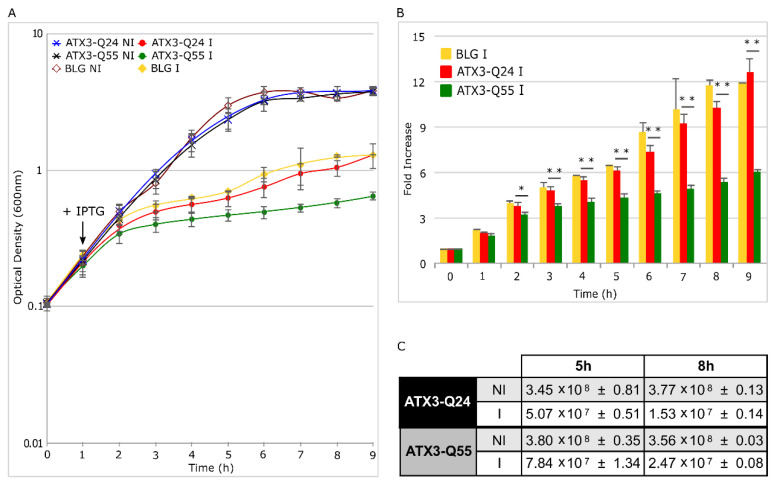
Effects of ataxin-3 (ATX3) variants expression on cell growth and viability. (**A**) Growth curves of *E. coli* Rosetta strains expressing ATX3-Q24 (black dots), ATX3-Q55 (light grey dots) and β-lactoglobulin (BLG) (grey squares). Cultures were inoculated at OD_600_ = 0.1 (t0), induced after 1 h with 0.4 mM of IPTG (t1, black arrow) and growth profiles were monitored at 37 °C for 8 h. As control, the same cultures (ATX3-Q24, black stars; ATX3-Q55, light grey stars; BLG, not filled grey squares) were inoculated without IPTG addiction (Not Induced, NI). Error bars represent standard deviations and are obtained from nine independent experiments. (**B**) Quantification of optical density increases. Bars represent the fold increases of *E. coli* Rosetta stains growth expressing ATX3-Q24 (black bars), ATX3-Q55 (light grey bars) and BLG (grey bars) normalized on t0 of each strain. Error bars represent error standard and are derived from nine independent experiments. Statistical analysis was made between BLG and ATX3-Q24 strains and did not show significance. ** *p*-value < 0.01, * *p*-value < 0.05. (**C**) Colony Formation Unit (CFU) count of ATX3-Q24 and ATX3-Q55 I and NI strains. At 5 and 8 h after IPTG induction, 1 mL of cell cultures were collected, normalized at OD_600_ = 0.5 and 1:10 serial dilutions were performed. Moreover, 100 µL of 10^−6^ and 10^−5^ dilutions were seeded on single LB agar plate supplemented with ampicillin 100 µg/mL and glucose 0.1%. After drying, colonies were let to growth over-night at 37 °C and then counted. Data were represented as CFU/mL with standard deviations of three independent experiments.

In our previous work [28], we demonstrated that the wild type ATX3 protein carrying 24 consecutive glutamines (ATX3-Q24) and the pathological ATX3 variant with 55 residues (ATX3-Q55) reproduce in *E. coli* cell the aggregation pathway observed in vitro and display different toxicity level; the pathogenic form, indeed, exhibits higher toxicity with respect to the wild type. To investigate the mechanisms at the basis of this difference, we first compared the growth curves of *E. coli* Rosetta-Gami strains expressing the ATX3-Q24 and the ATX3-Q55 variants, lacking any tag or fusion partner [28]. As a control, we employed the β-lactoglobulin (BLG), a non-amyloidogenic protein that aggregates into inclusion bodies (IBs) when overexpressed in *E. coli* [31]. Growth profiles were obtained by inducing protein expression after 1 h from the inoculum with 0.4 mM IPTG and curves were followed up to 8 h after induction. According to our previous work [28], the induced strains showed slower growth rates in comparison with the non-induced (NI) control strains (Figure 1A,B). The curves of the strains expressing ATX3-Q24 and BLG were quite similar, thus showing that the effect on cell growth exerted by wild type ATX3 is merely due to a metabolic burden effect caused by heterologous protein over-expression [32,33]. In contrast, the ATX3-Q55 growth curve was constantly slower than the physiological variant, up to two-fold lower at the end of the induction (t = 9 h, Figure 1B). In order to correlate the ATX3-Q55 toxicity to a decrease in cell viability, we analyzed the Colony Formation Unit (CFU) at 5 h and 8 h after induction. The results showed that after 5 h there were no differences in CFU count between the two induced strains and with respect to the relative NI strains. On the other hand, at 8 h after IPTG addition, both induced strains displayed a 10-fold lower CFU count than the NI strains, but there were no significant differences between them (Figure 1C).

Taken together, these results demonstrate that, although the expression of the two proteins have the same bactericidal effect on cell viability, the poly-Q expansion gives to the ATX3-Q55 pathological variant an additional bacteriostatic effect through mechanisms that will be further investigated.

### 2.2. ATX3-Q55 Toxicity Is Not Correlated to a Higher Protein Expression or Increase of the Soluble Fraction

**Figure 2:**
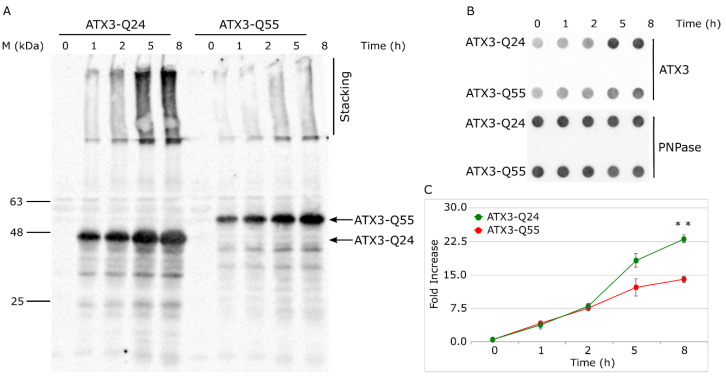
Analysis of ATX3 protein expression at different growth times. (**A**) SDS-PAGE (12%) and western blot analysis of protein total extracts (TE) obtained from *E. coli* Rosetta strains expressing ATX3-Q24 and ATX3-Q55. Cells were collected at different times after induction (t = 0, 1, 2, 5, and 8 h) and processed as detailed in Materials and Methods. Moreover, 20 μL of TE samples were boiled for 10 min and separated in SDS-PAGE, blotted onto Odyssey nitrocellulose membrane (LI-COR). ATX3 Z46 rabbit polyclonal antibodies [36] and IRDye fluorescent rabbit secondary antibody (LI-COR) was used for the immunodetection. Black bars indicate the migration of protein markers. Arrows represent the migrations of ATX3-Q24 and ATX3-Q55. The related gel stained with EZ-Blue Gel staining solution (Sigma) is reported in Figure S1. (**B**) Dot blot and (**C**) densitometric analysis of TE.5 μL of TE samples were diluted in 150 μL final volume of Sample Buffer. Then, were boiled 10 min and vacuum filtered in 48-well dot-blot apparatus through Odyssey nitrocellulose membrane (LI-COR). ATX3 variants and PNPase protein were revealed using anti-ATX3 Z46 and anti-PNPase rabbit polyclonal antibodies respectively [37]. Dots were quantified by densitometry and normalized using PNPase as a loading control and t0 signal of each strain as blank. Lines represent ATX3-Q24 (black dots) and ATX3-Q55 (light grey dots). Error bars represent standard error and derive from at least three independent experiments. ** *p*-value < 0.01.

To assess if the higher toxicity displayed by the ATX3 pathogenic form was due to a different protein expression, we quantified the amount of total ATX3 protein in ATX3-Q24 and ATX3-Q55 expressing strains by western blot. Equal amount of cells at different growth times (0, 1, 2, 5, and 8 h after induction) were collected and processed to obtain total protein extracts and samples were analyzed by SDS-PAGE (Figure S1) and western blot (Figure 2A). The results showed a different ATX3 expression level in the two strains; in particular, the ATX3-Q24 variant seems to be expressed at a higher level and forms more aggregate than the ATX3-Q55 protein. However, the presence of proteolytic fragments and aggregates in both strains did not allow an accurate quantification. For this reason, we performed dot-blot analysis to quantify the ATX3 protein level. The same samples used in western blot analysis were applied directly on a nitrocellulose membrane in a single spot and ATX3 signals were then quantified by immunofluorescence (Figure 2B,C). The data were obtained normalizing with respect to the PNPase content (a cytosolic protein constantly expressed at 37 °C and one of the most expressed proteins in *E. coli* [34,35]) and to the t0 signal of each strain. The results confirmed the highest ATX3-Q24 expression, with an increase of about 1.5-fold at 8 h (*p*-value < 0.01) after induction, with respect to the ATX3-Q55 (Figure 2C).

**Figure 3:**
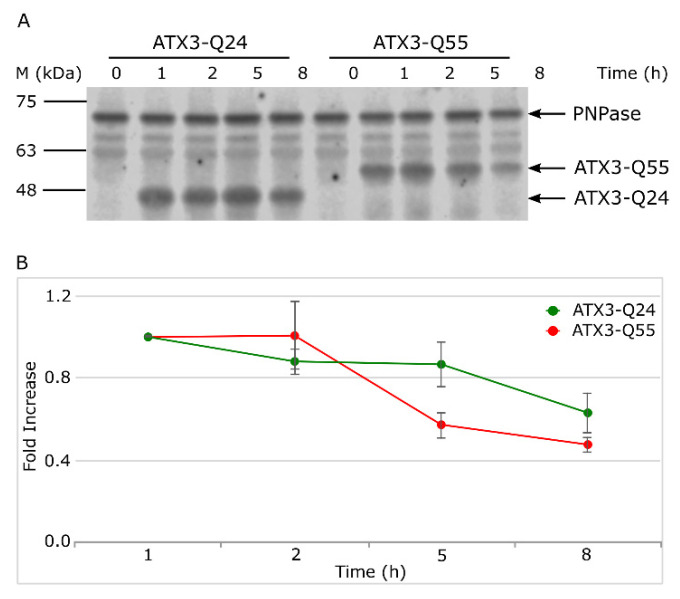
Analysis of soluble protein fraction of ATX3 variants. (**A**) Western blot of soluble fractions of *E. coli* Rosetta strains expressing ATX3-Q24 and ATX3-Q55. Soluble fractions were collected at different times after induction (t = 0, 1, 2, 5, and 8 h) and processed as detailed in Material and Methods. Black bars indicate the migration of protein markers. Arrows represent the migrations of ATX3-Q24, ATX3-Q55, and PNPase. (**B**) Western blot signals were quantified by densitometry and normalized respect to PNPase signals and t1 signal of each strain. Lines represent ATX3-Q24 (black dots) and ATX3-Q55 (light grey dots) trends. Error bars represent standard error and derive from at least three independent experiments.

Finally, we analyzed the soluble ATX3 fraction (Figure 3) to obtain information about the content of native and oligomeric species, which are SDS-soluble and migrate as monomeric protein in SDS-PAGE [28,38,39]. The quantification of western blot signals of monomeric band did not display significant differences between ATX3-Q24 and ATX3-Q55 soluble fractions, at any time of induction. Overall, the data demonstrate that ATX3-Q55 toxicity is not caused by a higher level of expression or amount of soluble fraction but argue the hypothesis of an intrinsic toxicity of ATX3-Q55 oligomeric species.

### 2.3. FTIR Analysis of E. coli Cell Modifications Induced by the Expression of ATX3-Q24 and ATX3-Q55

To explore in situ if the expression of the two ATX3 variants differently perturbed the host cells, we applied FTIR microspectroscopy to *E. coli* intact cells, at 5 and 8 h from protein induction. As a control, we also analyzed NI cells. To investigate the response of the main cell biomolecules, the FTIR analysis was performed in different spectral ranges. We analyzed the second derivatives of the FTIR spectra, which allow resolving the different overlapping components in the complex absorption bands. Moreover, to evaluate the reproducibility of the results and to identify the most significant spectral variations that enabled us to discriminate between induced and NI cells, we supported the FTIR data with a multivariate analysis approach, namely PLS-DA. Furthermore, to investigate if the expression of the two protein variants differently perturbed the host cells, the Euclidean distance between the PLS-DA scores was calculated between the induced and NI cells for each ATX3 variant.

For sake of clarity, in the following figures we reported the second derivatives of the representative FTIR absorption spectra identified by the multivariate analysis, as described in the Materials and Methods section. The average absorption and second derivative spectra with the standard deviation from the three independent experiments are shown in Figure S2. In Table S1 we reported—for all of the analyzed spectral ranges—the assignment of the relevant wavenumbers identified by PLS-DA.

#### 2.3.1. FTIR Study of Protein Secondary Structure Modifications: Analysis of Amide I Band

**Figure 4:**
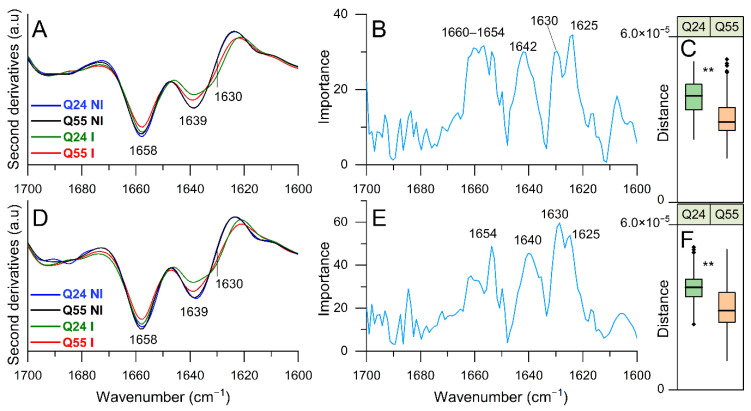
FTIR analysis of protein secondary structure modifications investigated in the 1700–1600 cm^−1^ spectral range. The second derivatives of representative FTIR spectra - identified by PLS-DA—of *E. coli* cells expressing ATX3-Q24 and ATX3-Q55 at 5 (**A**) and 8 (**D**) hours of induction, and of NI cells are reported. In (**B**) (5 h) and (**E**) (8 h), the loading plots representing the spectral components more relevant for the discrimination between the classes are displayed. In (**C**) (5 h) and (**F**) (8 h), the distribution of distances between I and NI is represented as box plots, where the horizontal line within the box is the median. The box ends show the first (Q1) and third quartiles (Q3), the upper whisker is the minimum between the absolute maximum and Q3 + 1.5* interquartile range (IQR), and the lower whisker is computed as the maximum value between the absolute minimum and Q1 − 1.5*IQR. Here, IQR is the interquartile range computed as Q3–Q1. Values beyond whiskers (outliers) are shown as dots. The distances are computed as following: the Euclidean distance between the PLS-DA scores was computed for each combination of samples within the two groups I and NI, obtaining an ensemble of distances for ATX3-Q24 and ATX3-Q55 independently. The horizontal bar above the two box plots shows the significance of the difference between the ATX3-Q24 and ATX3-Q55 distance distribution. The significance is computed using an unpaired two-sample T-test. **: *p*-value < 0.01. The reported spectroscopic analyses were performed on spectra from three independent experiments, where 5–7 spectra were collected for each condition in each experiment.

In Figure 4, we report the second derivative analysis of *E. coli* ATX3-Q24 and ATX3-Q55 induced cells and of NI cells at 5 h of induction, in the Amide I band, between 1700 and 1600 cm^−1^, arising mainly from the C=O stretching vibration of the peptide bond that provides information on the secondary structures [40] of the whole cell proteins.

In particular, the NI cell spectra were characterized by two main bands at ~ 1658 cm^−1^, due to α-helix and random coil structures, and at ~1639 cm^−1^, mainly assigned to native β-sheets [40]. After induction, in the ATX3-Q24 cell spectrum these components were found to decrease in intensity, and a shoulder appeared at ~1630 cm^−1^ that can be assigned to protein aggregates [29,41]. Similar features were observed for the ATX3-Q55 cell spectrum, where, however, the native β-sheet component displayed a minor decrease compared to ATX3-Q24, and the intermolecular β-sheet shoulder was less intense (Figure 4A). In addition, a reduction of the α-helix and random coil absorption was detected. Noteworthy, besides the component around 1660–1654 cm^−1^, assigned to α-helix and random coil structures (Figure 4B), the PLS-DA (Table S1) identified three distinct components of the β-sheet structures responsible for the discrimination between the analyzed classes: ~1642–1640 cm^−1^, due to native β-sheets, ~1630 cm^−1^ tentatively assigned to native-like β-sheets and β-sheets in protein aggregates, and ~1625 cm^−1^ due to intermolecular β-sheets [29,40,41]. Therefore, the shoulder at ~1630 cm^−1^ in the second derivative spectra is likely due to the overlapping absorption of two components, ~1630 cm^−1^ and ~1625 cm^−1^, as suggested by PLS-DA.

At 8 h of protein induction (Figure 4D), the spectral features of the induced cells were similar to those at the early time of induction. As reported in the loading plot of Figure 4E, the PLS-DA identified as relevant to the discrimination of the same main components found for 5 h of induction. All of this considered, the band at around 1639 cm^−1^ (Figure 4A,D), of higher intensity in cells expressing ATX3-Q55, could reflect the presence not only of native β-sheets, but also of native-like β-sheets of toxic species that, as we will illustrate in the following, could be responsible for the more important cell perturbation observed for the expanded variant compared to cells expressing ATX3-Q24.

The Euclidean distances between the PLS-DA scores of I and NI cells were found to be significantly different in the ATX3-Q55 and ATX3-Q24 expression for both the induction times. In particular, a higher distance was observed for ATX-Q24 compared to ATX-Q55 (Figure 4C,F) in agreement with the higher ATX3-Q24 expression detected by the biochemical analyses reported in the previous paragraph (Figure 2).

#### 2.3.2. FTIR Study of Lipid Modifications: Analysis of the 3050–2800 cm^−1^ Spectral Range

After the investigation of the differences in the whole cell protein secondary structure changes upon protein induction, we wondered whether the ATX3 expression could induce modifications in the membrane lipids and, in particular, if these changes could be different in cells expressing the two protein variants.

**Figure 5:**
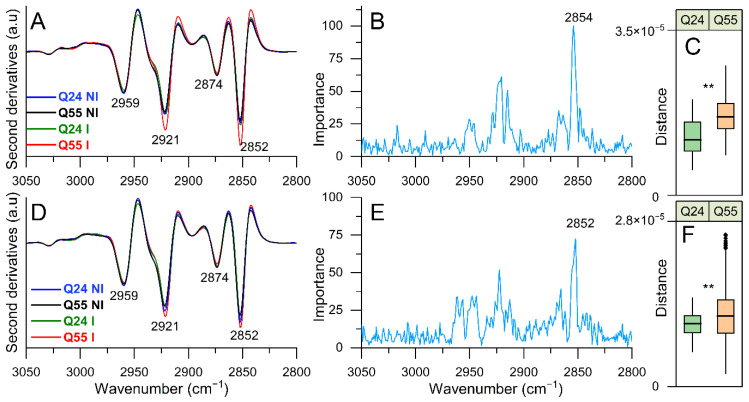
FTIR analysis of lipid modifications investigated in the 3050–2800 cm^−1^ CH_n_ stretching range. The second derivatives of representative FTIR spectra, identified by PLS-DA, of *E. coli* cells expressing ATX3-Q24 and ATX3-Q55 at 5 (**A**) and 8 (**D**) hours of induction, and of NI cells are reported. In (**B**) (5 h) and (**E**) (8 h), the loading plots representing the spectral components more relevant for the discrimination between the classes are displayed. In (**C**) (5 h) and (**F**) (8 h), the distribution of distances between I and NI is represented as box plots, as in Figure 4. The reported spectroscopic analyses were performed on spectra from three independent experiments, where 5–7 spectra were collected for each condition in each experiment. **: *p*-value < 0.01.

To this aim, in Figure 5A, we reported the second derivative spectra of ATX3-Q24 and ATX3-Q55 cells at 5 h of induction, and of NI cells, in the 3050–2800 cm^−1^ spectral range, dominated by the absorption of CH_n_ groups of lipid hydrocarbon chains. In particular, the spectra of NI cells are characterized by four main bands: ~2959 cm^−1^ (CH_3_ asymmetric stretching), ~2921 cm^−1^ (CH_2_ antisymmetric stretching), ~2874 cm^−1^ (CH_3_ symmetric stretching), and ~2852 cm^−1^ (CH_2_ symmetric stretching) [42]. In the induced cell spectra, particularly in ATX3-Q55, we observed an increase of the intensity of the CH_2_ bands at ~2921 cm^−1^ and 2852 cm^−1^ compared to NI cells. As displayed in the loading plot (Figure 5B), the ~2854 cm^−1^ component was identified by the PLS-DA as the most relevant to discriminate between the different sample classes. This result indicates an important variation of the chemico-physical properties of cell lipids in the ATX3-Q55 cells, where the CH_2_ band intensity was significantly greater compared to ATX3-Q24 cells (Figure 5A,C). In particular, the lipid content of cells expressing the expanded ATX3 variant could be characterized by longer hydrocarbon chains that would lead to a variation of membrane fluidity and permeability [43]. In addition, this result could also reflect the acetylation of lipid A, a component of cell membrane lipopolysaccharide (LPS), as response to a stress that is counteracted for promoting cell survival [44].

At 8 h of protein induction (Figure 5D–F), a quite similar spectral behavior was detected, even if the differences between ATX3-Q55 and NI cells are less important compared to the early time of induction. In this spectral range, the Euclidean distances between the PLS-DA scores of I and NI cells were found to be significantly higher in the ATX3-Q55 expressing cells compared to ATX3-Q24 (Figure 5C,F), indicating more important lipid modifications induced by the expanded variant.

#### 2.3.3. FTIR Study of Lipid and Peptidoglycan Modifications: Analysis of the 1500–1200 cm^−1^ Spectral Range

**Figure 6:**
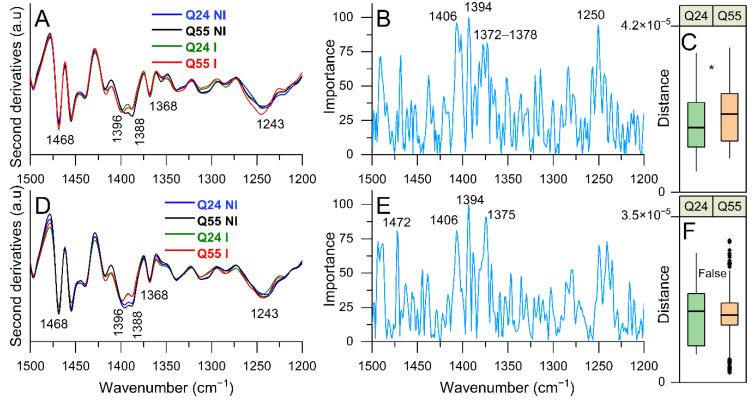
FTIR analysis of the 1500–1200 cm^−1^ spectral range. The second derivatives of representative FTIR spectra, identified by PLS-DA, of *E. coli* cells expressing ATX3-Q24 and ATX3-Q55 at 5 (**A**) and 8 (**D**) hours of induction, and of NI cells are reported. In (**B**) (5 h) and (**E**) (8 h), the loading plots representing the spectral components more relevant for the discrimination between the classes are displayed. In (**C**) (5 h) and (**F**) (8 h), the distribution of distances between I and NI is represented as box plots, as in Figure 4. The reported spectroscopic analyses were performed on spectra from three independent experiments, where 5–7 spectra were collected for each condition in each experiment. *: *p*-value < 0.05.

In Figure 6A, the second derivative spectra of ATX3-Q24 and ATX3-Q55 cells at 5 h of induction, and of NI cells, are reported in the spectral range between 1500 and 1200 cm^−1^, mainly due to the overlapping absorption of lipids, peptidoglycans, and phosphates, thus giving information on possible modifications of the cell envelope properties [45,46].

In particular, the second derivative spectra of NI cells are characterized by a band at ~1468 cm^−1^ due to CH_2_/CH_3_ deformations of lipid hydrocarbon chains, two components at ~1396 cm^−1^ and at ~1388 cm^−1^ assigned mainly to CH_2_/CH_3_ of lipid polar heads and hydrocarbon chains, as well as of peptidoglycans [42,45,46]. Moreover, an absorption occurred at ~1368 cm^−1^, due to the overlapping absorption of CH_2_ groups of lipids and of acetylglucosamine in peptidoglycans and in lipopolysaccharides (including lipid A) [42,45,46]. Then, a component at ~1243 cm^−1^, due to phosphate groups (PO_2_- antisymmetric stretching), has been also detected [42,45]. In agreement with what observed between 3050 and 2800 cm^−1^, the CH_2_/CH_3_ band at ~1468 cm^−1^ was found to slightly increase in intensity in ATX3-Q55 cells compared to ATX3-Q24 and NI cells. Furthermore, the ~1396 cm^−1^ band was found to upshift at ~1398 cm^−1^ in the induced cells expressing the two ATX3 variants. Overall, these results suggest a reorganization of the structure and/or composition of peptidoglycans and lipids, more evident in ATX3-Q55 cells, as also supported by the phosphate absorption at ~1243 cm^−1^ that in the ATX3-Q55 cells was upshifted to ~1246 cm^−1^ and appears more intense compared to ATX3-Q24 and NI cells. We should note that the assignment of this band is not unequivocal, being due to phosphates, typical for instance of phospholipids as well as of nucleic acids [42,47]. Moreover, it could be assigned to phosphate groups of *E. coli* lipid A: indeed, the substitution of the lipid A sugar moieties by phosphate groups is known to mediate cell resistance to stress factors, affecting in particular the physical properties of the outer membrane [44,48,49]. In this regard, we should note that, since the PLS-DA did not identify as relevant for the discrimination other important spectral markers of nucleic acids, and the spectral variations of the phosphate band and of the main lipid moieties occurred simultaneously (see also Figure 5), the phosphate absorption at ~1243 cm^−1^ could be tentatively assigned to phosphorylation events and/or variation of the phospholipid content.

In addition (Table S1), the PLS-DA identified as relevant for the discrimination also the component at around 1406 cm^−1^ that could be assigned to lipid polar heads CH_3_ [42]. In this spectral range, and at 5 h of induction, the Euclidean distances between the PLS-DA scores of I and NI cells were found to be significantly higher in the ATX3-Q55 expressing cells compared to ATX3-Q24 (Figure 6C), indicating again more important spectral modifications induced by the expanded variant. At 8 h of induction (Figure 6D–F), the spectral differences between ATX3-Q24 and ATX3-Q55 expressing cells resulted not significant.

#### 2.3.4. FTIR Study of Lipopolysaccharide and Peptidoglycans: Analysis of the 1200–900 cm^−1^ Spectral Range

The fingerprint range between 1200–900 cm^−1^ is due to the complex absorption of different biomolecules, including carbohydrates and phosphates, and in the case under consideration, the observed IR response is ascribable mainly to lipopolysaccharide and peptidoglycan moieties [29,42,46]. Moreover, in this case, we will discuss only the spectral components relevant for the discrimination between the different sample classes, as resulted from PLS-DA (Table S1).

**Figure 7:**
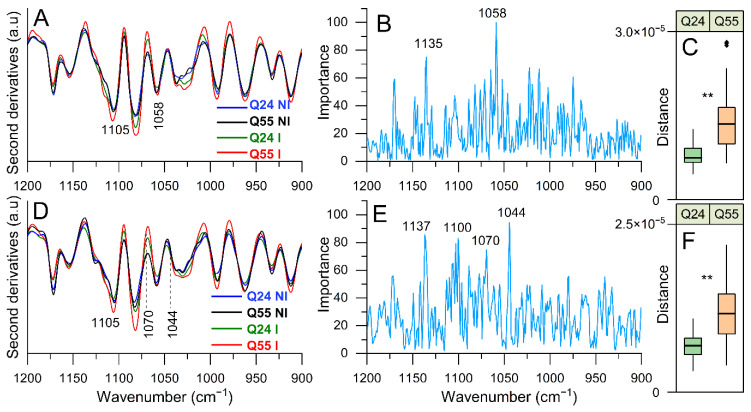
FTIR analysis in the 1200–900 cm^−1^ spectral range. The second derivatives of representative FTIR spectra, identified by PLS-DA, of *E. coli* cells expressing ATX3-Q24 and ATX3-Q55 at 5 (**A**) and 8 (**D**) hours of induction, and of NI cells are reported. In (**B**) (5 h) and (**E**) (8 h), the loading plots representing the spectral components more relevant for the discrimination between the classes are displayed. In (**C**) (5 h) and (**F**) (8 h), the distribution of distances between I and NI is represented as box plots, as in Figure 4. The reported spectroscopic analyses were performed on spectra from three independent experiments, where 5–7 spectra were collected for each condition in each experiment. **: *p*-value < 0.01.

In agreement with what observed in the other spectral ranges, the spectral profile of the NI cells is different from that of ATX3-Q24 and ATX3-Q55 expressing cells at 5 and 8 h of induction (Figure 7A,D). Again, the spectral differences were more pronounced for ATX3-Q55 cells (Figure 7C,F) confirming in particular the results obtained between 1500 and 1200 cm^−1^. As indicated by the multivariate analysis (Figure 7B,E), among the components carrying the higher discrimination capacity we found the ~1058 cm^−1^ and the complex absorption between ~1137 and 1100 cm^−1^, with a main peak at 1105 cm^−1^ (Figure 7A), that appears of higher intensity in ATX3-Q55 cells. Moreover, at 8 h of induction (Figure E), PLS-DA identified also the components at ~1044 cm^−1^ (that appears as a shoulder of the band at ~1039 cm^−1^) and at ~1070 cm^−1^ (delimiting the peak at ~1058 cm^−1^ in the second derivative spectra).

Overall, all of these IR bands are ascribable to complex vibration modes of carbohydrates, mainly of peptidoglycans, and to phosphate groups that we mainly assigned—as we discussed before—to some components of the cell envelope, including the lipopolysaccharide barrier [42,45,46].

### 2.4. Electron Microscopy Analysis of ATX3 Expressing Strains

**Figure 8:**
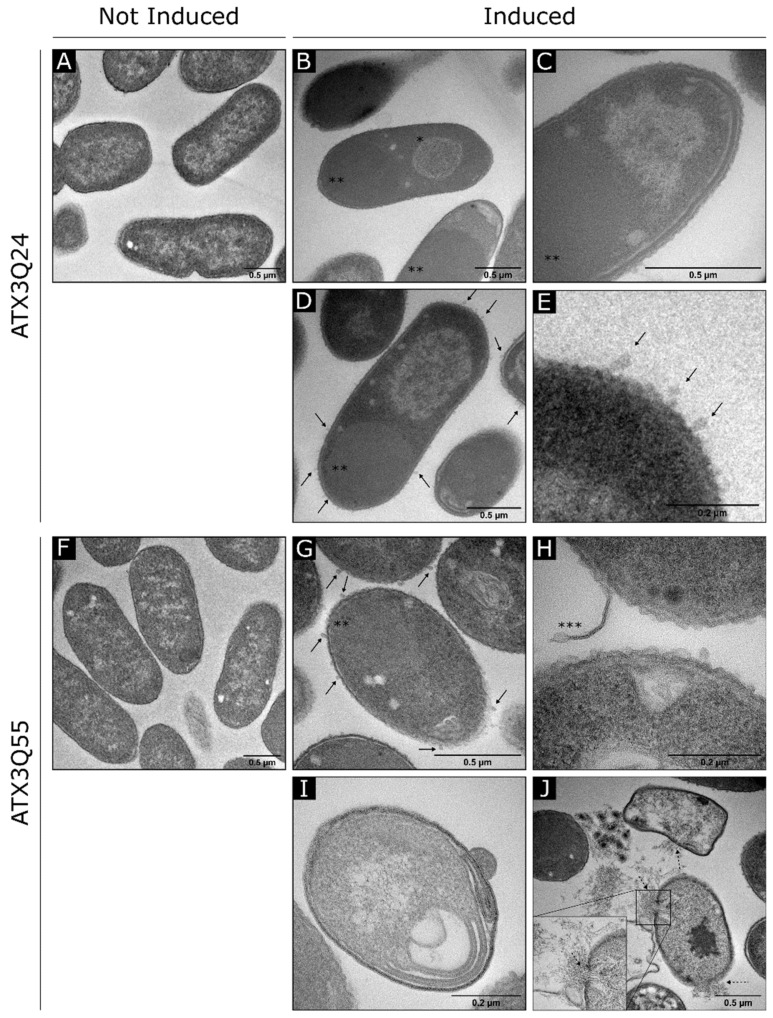

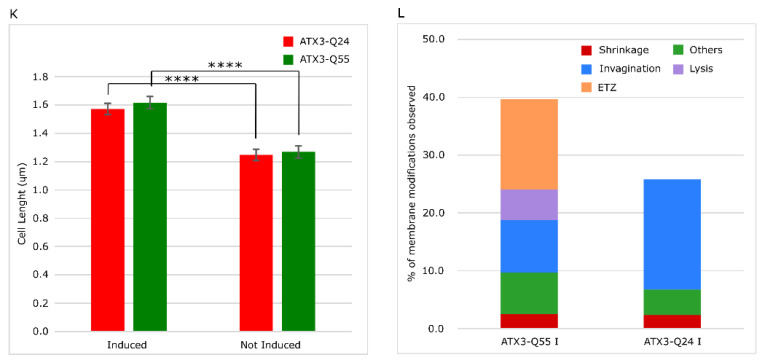
Transmission Electron Microscopy micrographs and analysis of *E. coli* ATX3-Q24 and ATX3-Q55 expressing strain; 2 OD of induced and NI cells were collected at 5 h of induction and treated as reported in materials and methods (par. 4.7). (**A**) ATX3-Q24 not induced cells. (**B–E**) ATX3-Q24 induced strain. (**F**) ATX3-Q55 not induced strain. (**G–J**) ATX3-Q55 induced strain. IB, inclusion body. Arrows indicate vesicles. * DNA condensation. ** Cytoplasmatic IBs. *** Outer membrane detachment. Dashed arrows indicate membrane destruction and cell lysis. (**K**–**L**) Cell length and classification of membrane alterations of *E. coli* Rosetta strains expressing ATX3-Q24 (black bars), ATX3-Q55 (light grey bars). Cell growth 5 h after IPTG addition (I), or not (NI), were prepared for thin-layer EM microscopy. Cell length (K) was automatically measured after TEM imaging using ImageJ software. Significant differences among groups (ATX3-Q24 NI, ATX3-Q55 NI, ATX3-Q24 I, ATX3-Q55 I) were assessed by factorial analysis of variance (ANOVA) followed post-hoc Tukey’s HSD test. Error bars represent error standard (*n* = 333). **** *p* < 0.0001. Membrane alterations (L) were classified as shrinkage (red), invaginations (blue), electron transparent zone (ETZ; orange), lysis (light violet) and not distinguishable (ND, not distinguishable between shrinkage and invagination; green). Data are expressed as percentage on total cells analyzed (*n* = 333).

Transmission Electron Microscopy was employed to analyze cellular ultrastructure of the *E. coli* expressing the ATX3 variants (Figure 8). As expected [50], we observed a cell elongation of the induced strains, with a mean length of 1.571 µm and 1.616 µm for ATX3-Q24 and ATX3-Q55, respectively, in comparison with NI strains values that are both 25% shorter than induced strain (*n* = 333 for both the strains; Figure 8K). A particular feature of a population of ATX3-Q24 induced cells is the presence of condensed DNA (Figure 8B, single asterisk). Interestingly, this kind of DNA structure was observed in bacteria under different stress conditions, like nutritional stress [51,52,53]. Cytoplasmic IBs (Figure 8G, double asterisk) and production of extra-cytoplasmic vesicles (arrows) were visible in both over-expressing strains, as a response of intracellular protein accumulation. Other common characteristics of the induced cells are the shrinkage and the formation of inner membrane invaginations (Figure 8C,I,L). Proliferation of inner membrane has been reported for cells expressing membrane proteins [50,54,55]. Noteworthy, specific alterations i.e., formation of large electron transparent zones, detachment of the outer membrane and cell lysis were observed only in ATX3-Q55-expressing cells (Figure 8H, triple asterisk, Figure 8I,J,L), highlighting the higher toxicity of the expanded protein.

## 3. Discussion

Hallmark events in neurodegenerative disorders are the misfolding, the aggregation and the accumulation of proteins, which are responsible for cellular dysfunction, loss of synaptic connections, and brain damage [1,2,3]. Even if specific proteins are involved in the different diseases, the process of protein misfolding and aggregation is remarkably similar. The aggregation process is generally triggered by protein misfolding, followed by soluble oligomeric aggregates formation, which in turn are precursors to fibrils [4]. Many evidences underlined the pivotal role of soluble oligomeric species in triggering cellular toxicity [15,16,17]. Several potential mechanisms underlie oligomer toxicities, including neuron membrane disruption by increase in membrane conductance or leakage in the presence of small globulomers to large prefibrillar assemblies, direct formation of ion channels, binding to different cell-surface receptors, and oxidative stress [17,18,19]. To these effects are added those deriving from the loss of the activity of the protein associated with each particular disease. In this context, the possibility to investigate the aggregation pathway in a simple transgenic system as *Escherichia coli* offers the opportunity to study the relationship between fibrillogenesis and toxicity. In a previous work, we characterized for the first time in the intracellular environment of *E. coli* the multistep aggregation pathway of the poly-Q protein ataxin-3 (ATX3). We specifically investigated the correlation between aggregation and cytotoxicity, and we detected the toxic effect of the oligomeric species [28]. Using the same *E. coli* strains, we tried to elucidate the mechanisms underlying the pathological phenotypes in the present work. At first, we confirmed the greater detrimental effect of the expanded ATX3 expression (ATX3-Q55) with respect to the wild type (ATX3-Q24) on cell growth (Figure 1A,B). We also provided additional data supporting the bacteriostatic effect of toxic species of ATX3-Q55, as substantiated by CFU assays (Figure 1C,D). The less toxic variant (the physiological ATX3-Q24) was found to display the highest level of expression and of aggregated species compared to the expanded ATX3-Q55 protein (Figure 2), allowing to discard a significant impact of the insoluble fraction in the observed differential cytotoxicity. It is possible that the comparable protein level presented in the soluble fraction of the two strains could be the consequence of the aggregate sequestration in the IBs. These results were further supported by the FTIR analysis: in the spectral range sensitive to the protein secondary structures and aggregation (Amide I band), the spectral differences between NI and induced cells were more evident in ATX3-Q24 cells compared to those expressing the expanded variant, at both times of protein induction (Figure 4). In particular, the most important differences were due to the β-sheet content, with three components identified by the PLS-DA: the ~1625 cm^−1^ (β-sheets typical of insoluble protein aggregates), the ~1630 cm^−1^ (native-like β-sheets and β-sheets of protein aggregates), and the ~1642 cm^−1^ (native β-sheets) (Figure 4B,E). Furthermore, the component at 1654–1660 cm^−1^, assigned to α-helix/random coil structures, resulted relevant for the classification (Figure 4B,E). Noteworthy, the β-sheet component typical of insoluble aggregates (IBs) displayed a higher intensity in the ATX3-Q24 compared to the ATX3-Q55 (Figure 4A), while the native/native-like β-sheet components appeared of higher intensity in the expanded form compared to ATX3-Q24. Considering that, as we will discuss below, the expanded form was responsible for the most important changes in the other spectral ranges compared to the ATX3-Q24 expression, we can speculate that the component assigned to native-like β-sheets could reflect the presence of soluble protein assemblies, which we may assume to be oligomers, responsible for the observed cell perturbation. Concerning this point, we should note that the early aggregated forms of the Josephin domain were found to display a native-like structure, as detected by nano-IR spectroscopy [7]. On the other hand, the component due to IB formation, at 5 h of protein induction displayed a higher intensity in ATX3-Q24 compared to ATX3-Q55 (Figure 4A), supporting that the major role in cell perturbation cannot be ascribed to insoluble protein embedded in IBs.

As reported in the literature, the overexpression of recombinant proteins could represent a stress for the host cells that increases in the case of misfolded-prone proteins. In such a case, to counteract the effects of toxic species, cells overexpress chaperones and a number of non-heat shock genes [56]. Interestingly, protein overexpression modulates also the abundance of some membrane proteins, with possible consequences in cell trafficking and signaling [57]. Moreover, the expression of a hyper-amyloidogenic protein (the A31V variant of RepA-WH1) was found to alter, in turn, the expression and function of several proteins involved in cell response to stress. In particular, the identified proteins are involved in cell processes—including the transport through membranes, iron uptake, detoxification of hydrogen peroxide, and respiration—that could be affected by membrane integrity. Therefore, the alteration of these vital processes, due to the presence of protein toxic species, implies a modification of the chemico-physical and functional properties of cell membranes [58].

A direct involvement of *E. coli* cell lipid modifications was described as consequence of recombinant protein expression [43]. In our previous work, we detected changes in membrane fluidity, cell permeability, and lipid composition that correlated with the presence of soluble aggregates. This was possible by monitoring the temporal evolution of protein aggregation that showed important modifications in cell lipids when the protein aggregates were soluble, and not once the overexpressed protein becomes immobilized inside IBs [43]. In the present work, in addition to the analysis of the spectral range mainly due to the absorption of the lipid hydrocarbon chains (3050–2800 cm^−1^), we extended the study to other spectral ranges where not only lipids, but also peptidoglycans absorb, providing information on the modifications of the properties of the cell envelope biomolecules. As described in literature, the “cell envelope stress response system” (ESRS) monitors processes that are mediated by the cell wall and membranes, and counteracts the effects of several stressors, serving as a permeability barrier to protect cells from adverse conditions [44,59,60,61]. Noteworthy, our FTIR results indicate that misfolded proteins elicit modifications in cell envelope not only at the level of the lipid membranes, but also in the peptidoglycans and LPS barriers. We found that cells expressing ATX3-Q55 displayed the most important spectral differences, indicating in particular significant modifications of lipid and peptidoglycan properties. Indeed, the observed increase of the intensity of the hydrocarbon chain CH_2_ (Figure 5A), particularly evident at 5 h of protein induction, could reflect an elongation of the lipid hydrocarbon chains, impacting on membrane fluidity and permeability. Together with the simultaneous increase of the intensity of phosphate and carbohydrate moieties between 1300 and 900 cm^−1^ (Figure 6 and Figure 7), this result could be ascribed to a rearrangement of glycerophospholipid composition, which is a crucial adaptation response to different stresses in bacteria [48]. Moreover, these spectral changes could also reflect modifications of lipid A, that is a key component of LPS, and it is known to undergo extensive remodeling, consisting in modification of acyl chains, such as palmitoylation, and in phosphorylation [44].

These results were confirmed by the analysis of the spectral range between 1500 and 1200 cm^−1^ where, in addition, induced cells displayed significant modifications of few bands that are assigned specifically to peptidoglycans, as well as to complex carbohydrate moieties that could be also ascribed to lipopolysaccharides [42,45,46]. Again, we should stress that more extensive spectral changes have been observed in cells expressing the expanded ATX3 variant (Figure 6).

Then, the significant increase of phosphate moieties that could reflect phospholipid modifications as well as phosphorylation changes of lipid A supports the important variation borne by the cell envelope whose integrity is, therefore, likely affected by the presence of soluble aggregates. Noteworthy, the analysis of the fingerprint region, between 1200 and 900 cm^−1^, dominated by the peptidoglycan absorption [45], strengthens these results, highlighting again the involvement of the main cell envelope macromolecule modifications as a response to the stress induced by the presence of toxic soluble forms of ATX3-Q55 (Figure 7).

Finally, ultrastructural modifications were observed in the *E. coli* expressing both ATX3 variants, some of which are due to the protein over-expression, such as the cell elongation [50], the formation of cytoplasmic IBs [62] and the production of extra-cytoplasmic vesicles [54] (Figure 8). It was demonstrated that the production of outer membrane vesicles is a general and independent mechanism of stress response in gram-negative bacteria that allows the removal of misfolded proteins accumulated in the envelope, and thus increases cell survival [50,54,55]. Noteworthy, the most drastic phenotype has been observed in the ATX3-Q55 strain, where the protein expression induced not only invagination of the inner membrane and shrinkage, as observed in the wild type strain (Figure 8C,I,L), but also the formation of large electron transparent zones, detachment of the outer membrane and cell lysis (Figure 8H, double asterisk; Figure 8I,J,L). This effect agrees with the previously demonstrated capacity of ATX3-Q55 oligomers to bind and damage membranes causing cell lysis in eukaryotic cells [17].

## 4. Materials and Methods

### 4.1. Bacterial Strain and Plasmid

In this work, we used the *E. coli* Rosetta-GamiTM-2 (DE3) (Novagen, Temecula, CA, USA) as expression strain. This strain is engineered to enhance expression of proteins having codons rarely used in *E. coli*, thanks to the presence of the pRARE2 plasmid that encode genes for seven rare tRNAs. Moreover, this strain carries the trxB and gor mutations that improve disulfide bond formation in the cytoplasm. Rosetta-GamiTM-2 was transformed with the pET21A-ATX3-Q24 and pET21A-ATX3-Q55 plasmids that have been obtained by cloning the two ATX3 genes into pET21A vector (EMD Biosciences, Dramstadt, Germany) between the NdeI/XhoI restriction sites [28].

### 4.2. Growth Curves Analysis

*E. coli* Rosetta strains expressing ATX3 variants were growth in LB medium (10 g/L peptone, 5 g/L yeast extract, 10 g/L NaCl), supplemented with ampicillin 100 μg/mL and glucose 0.1%. Growth experiments were performed at 37 °C, under constant shacking of 160 rpm. Cells were inoculated at OD_600_ = 0.1 dilution and induced with 400 μM IPTG when the cultures reach the OD_600_ = 0.2. After induction, growth profiles were measured by monitoring OD_600_ at each hour after induction for 8 h. At t0—1—2—5 and 8 h after induction, cell samples were normalized at the same OD and collected by centrifugation at 7000× *g*, 10 min at 4 °C to analyze protein content by Western blot. At the same times, cell samples were harvested to obtain sample for FTIR analysis (see below).

### 4.3. Colony-Formation Unit (CFU) Count

At 5 and 8 h after IPTG induction, 1 mL of cell cultures were collected, normalized at OD_600_ = 0.5 and 1:10 serial dilution was performed. 100 µL of 10^−6^ and 10^−5^ dilutions were seeded on LB agar plates supplemented with ampicillin 100 µg/mL and glucose 0.1%. After drying, colonies were let to growth overnight at 37 °C and then counted.

### 4.4. Western Blot Analysis of Total Extract and Soluble Protein Fractions

Total extract (TE) and soluble protein fraction (SP) samples from cell cultures collected at different growth times were lysed by resuspension in 300 μL of Lysis Buffer (PBS, pH 7.2, 20 mM, imidazole 10 mM, glycerol 10%, PMSF 2 mM, β-mercaptoethanol 1 mM, lysozyme 1 mg/mL, SIGMAFAST™ protease inhibitor cocktail (Sigma-Aldrich, St. Louis, MO, USA), incubated 10 min at room temperature under shacking condition and then sonicated 10 s at 10% amplitude. 100 μL of each sample were collected and the remaining 200 μL were centrifuged for 10 min at 20,000× *g* at 4 °C and supernatant (represented SP fraction) were collected. Sample buffer (Tris-HCl pH 6.8 0,25 M, SDS 2%, glycerol 20%, β-mercaptoethanol 0.708M, Bromophenol blue) was added in both TE and SP samples.

For Western blot analysis, 20 μL of both TE and SP samples were boiled for 10 min and used in SDS-PAGE on 12% resolving gel. Then, the gel was blotted on Odyssey nitrocellulose membrane (LI-COR, Lincoln, NE, USA) and ATX3 variants and PNPase proteins were revealed using anti-AT3 Z46 (1:5000) rabbit polyclonal and anti-PNPase (1:150,000) rabbit polyclonal antibodies [36,37]. We employed PBS plus 5% milk as blocking solution. Immunoreactive bands were detected with IRDye fluorescent rabbit secondary antibody (1:15000; LI-COR, Lincoln, NE, USA). When required, protein bands fluorescence intensity was quantified using Image Studio™ software (LI-COR, Lincoln, NE, USA).

### 4.5. Dot-Blot Analysis of Total Extract Samples

The 5 μL of TE samples were diluted in 100 μL final volume of Sample Buffer, and then were boiled for 10 min and vacuum filtered in 48-well dot-blot apparatus through Odyssey nitrocellulose membrane (LI-COR, Lincoln, NE, USA). ATX3 variants and PNPase proteins were revealed using anti-AT3 Z46 (1:5000) rabbit polyclonal and anti-PNPase (1:150,000) rabbit polyclonal antibodies. Dot density was quantified using Image Studio™ software (LI-COR, Lincoln, NE, USA).

### 4.6. FTIR Microspectroscopy Analysis

The 2.5 OD of Rosetta cell cultures were collected at the time indicated above, centrifugated for 2 min at 1000× *g* at 4 °C, and washed two times in physiological solution (0.9% NaCl *w/v* in sterile Milli-Q water).

Then, cell pellets were resuspended in a few microliters of 0.9% NaCl, and about 2 μL of the cell suspension were deposited onto a BaF2 window and dried at room temperature for about 30 min to eliminate the excess of water.

FTIR absorption spectra were acquired in transmission mode, in the 4000–700 cm^−1^ spectral range, by a Varian 610-IR infrared microscope coupled to the Varian 670-IR FTIR spectrometer (both from Varian Australia Pty Ltd., Mulgrave, VIC, Australia), equipped with a mercury cadmium telluride nitrogen-cooled detector. The variable microscope aperture was adjusted to ~200 μm × 200 μm. Measurements were performed at 2.0 cm^−1^ spectral resolution; 25 KHz scan speed, triangular apodization, and by the accumulation of 512 scan co-additions.

Spectra were corrected—when necessary—for the residual water vapor absorption by subtraction of the vapor spectrum. For comparison the spectra were normalized at the Amide I band area and the second derivative analysis was performed—after a 13-point smoothing of the measured spectra—by the Savitzky–Golay method (3rd polynomial, 13 smoothing points), using the GRAMS/32 software (Galactic Industries Corporation, Salem, NH, USA).

For each sample, we repeated several measurements by selecting different areas on the same sample (5–7 spectra for each condition in each experiment) through the variable diaphragm aperture of the infrared microscope. Furthermore, to evaluate the reproducibility of the results, we performed three independent experiments.

### 4.7. Multivariate Analysis of the FTIR Data

Multivariate analysis of the FTIR data has been performed using R version 3.6.3. In the preprocessing procedure, raw spectra have been checked for outliers using a fast algorithm for identifying multivariate outliers in high-dimensional datasets, as described in [63] and implemented in the R package mvoutlier version 2.0.5. Since no outliers were found, all spectra were retained for further analyses. Spectra have been split into four spectral ranges (3050–2800, 1700–1600, 1500–1200, 1200–900 cm^−1^) and on each range partial least square discriminant analysis (PLS-DA) was applied. The analysis has been performed on the second derivative spectra.

PLS-DA is a widely used multidimensional regression method, which is a variant of the classical partial least square method when the dependent variable is categorical [64].

Samples were partitioned into different subsets (see results), and different models were trained on each subset. In particular, two different models have been trained separately by splitting the spectra, based on the induction time.

In order to assess the predictive discrimination and avoid over-fitting, for each subset a 3-time repeated 10-fold cross-validation was applied; in this way, for each subset, 30 models were trained. The best model has been selected using the “one standard error rule”. In this case, the model with the best performance value is identified and, using resampling, we can estimate the standard error of performance. The final model used was the simplest model within one standard error of the (empirically) best model [65]. The classification performance was evaluated by the accuracy, i.e., the proportion of true results (true positive + true negative) over the total number of samples. The wavenumber importance was measured based on weighted sums of the absolute PLS-DA regression coefficients. The importance value was then normalized within the range 0–100.

The distribution of distances between I and NI for ATX3-Q24 and ATX3-Q55 has been obtained by computing the Euclidean distance between all pairs of spectra (in the low dimensional PLS score space), between group I and NI for ATX3-Q24 and for ATX3-Q55, which is:

(1) $$D{\left(K\right)}_{i,j}\;=\;\frac{1}{L}\;\sqrt{\sum_{c=1}^{c}(x_{i}^{c}\;-\;x_{j}^{c}{)}^{2}}$$

where *i* is the *i*-th spectra belonging to the I group, while *j* is the *j*-th spectra belonging to the NI group. *L* is the number of PLS scores and *c* is the *C*-th PLS component. *K* is either ATX3-Q24 or ATX3-Q55. In order to assess the difference between the distances in ATX3-Q24 and ATX3-Q55 a two sample T-test has been performed.

In Figure 4, Figure 5, Figure 6 and Figure 7, the second derivatives of the representative spectra, identified by selecting the spectrum having the minimum average (arithmetic average) distance (Euclidean metric) between all other spectra in the low-dimensional PLS scores space, have been shown.

### 4.8. Transmission Electron Microscopy Analysis

The 2 OD of ATX3Q24 and ATX3Q55 induced and NI cells were collected after 5 h of induction by centrifugation (10 min at 7200× *g* at 4 °C), washed with 0.5 mL of cold and filtered PBS and then fixed for 1 h with 1.5% glutaraldehyde solution in 0.1 M Phosphate Buffer (PB) at room temperature on a shaker. After centrifugation, the pellet was fixed for another hour in 1% glutaraldehyde in 0.1 M PB and subsequently post-fixed in 1% Osmium tetroxide solution prepared in the same buffer. After several washes, samples were stained overnight at 4 °C in 1% Uranyl Acetate aqueous solution, dehydrated in a series of alcohols and finally transferred in Propylene Oxide. Subsequently, samples were infiltrated in graded propylene oxide-epon epoxy resin, and finally embedded in resin. After 2 days hardening in oven at 65 °C, samples were sectioned in 70 nm slices by means of Reichert-Jung Ultracut E ultramicrotome. Transmission Electron Microscope (TEM) micrographs were acquired by means of JEOL JEM-2100Plus TEM (JEOL, Akishima, Tokio, Japan) operating with an acceleration voltage of 200 kV, and equipped with an 8 megapixel Gatan (Gatan, Pleasanton, CA, USA) Rio™ complementary metal-oxide-semiconductor (CMOS) camera.

## 5. Conclusions and Future Perspectives

In this work, we compared the aggregation of physiological and pathological expanded ATX3 proteins in the intracellular environment of *E. coli.* We showed that, although ATX3-Q24 variant forms insoluble aggregates at greater extent compared to ATX3-Q55, it is the pathological variant that exerts more toxic effects, affecting in particular the components of the cell envelope. This impact is likely connected with the soluble/toxic aggregates formed by the expanded variant, in agreement with what was found in more complex cell systems, where oligomers have been shown to trigger several toxic pathways, including severe membrane damages and oxidative stress.

The use of *E. coli* cells as simple model system allowed us to detect by FTIR microspectroscopy, by EM, and by biochemical assays the cellular effects of different protein aggregates formed in vivo, a challenging goal considering that protein aggregates are dynamic structures. Moreover, the possibility to correlate specific protein species with cellular toxicity could make it possible to develop a new screening method to test the efficacy and the mode of action of potential anti-amyloidogenic compounds in vivo.

## Data Availability

Data is contained within the article or supplementary material.

## Supplementary Materials

The following are available online at https://www.mdpi.com/1422-0067/22/2/943/s1, Figure S1: SDS-PAGE (12%) of protein total extracts (TE) obtained from *E. coli* Rosetta strains expressing ATX3-Q24 and ATX3-Q55, Figure S2: Absorption (top panel) and second derivative spectra (bottom panels) of *E. coli* cells expressing ATX3-Q24 and ATX3-Q55 at 5 and 8 h of induction, and of NI cells, Table S1: Peak position and assignment of the main spectral components identified by PLS-DA.
