# Supplementary material for: Pathological ATX3 Expression Induces Cell Perturbations in E. coli as Revealed by Biochemical and Biophysical Investigations
Source: Int J Mol Sci. 2021 Jan 19;22(2):943. doi: 10.3390/ijms22020943 (PMC7835732; doi:10.3390/ijms22020943)
Supplement: Supplementary file 1 [file ijms-22-00943-s001.pdf]

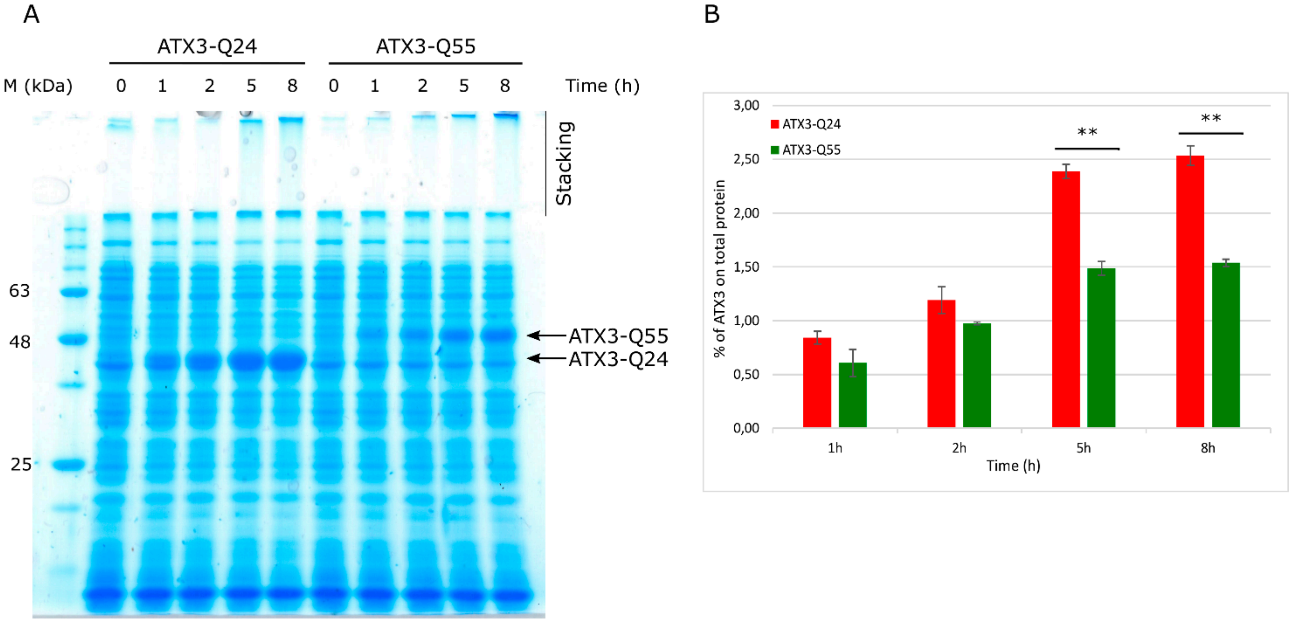

Figure S1: SDS-PAGE (12%) of protein total extracts (TE) obtained from *E. coli* Rosetta strains expressing ATX3-Q24 and ATX3-Q55. (**A**) Cells were collected at different times after induction (t=0, 1, 2, 5 and 8 h) and processed as detailed in Materials and Methods. 20 µL of TE samples were boiled for 10 min, separated in SDS-PAGE and stained with EZBlue Gel Staining Reagent (Sigma). Arrows represent the migrations of monomeric full-length ATX3-Q24 and ATX3-Q55. (**B**) Monomeric full-length protein signals at the different growth times were quantified by densitometry and expressed as percentage of the total amount of the proteins for each line. Error bars represent standard error and derive from two independent experiments. ** p-value < 0.01.

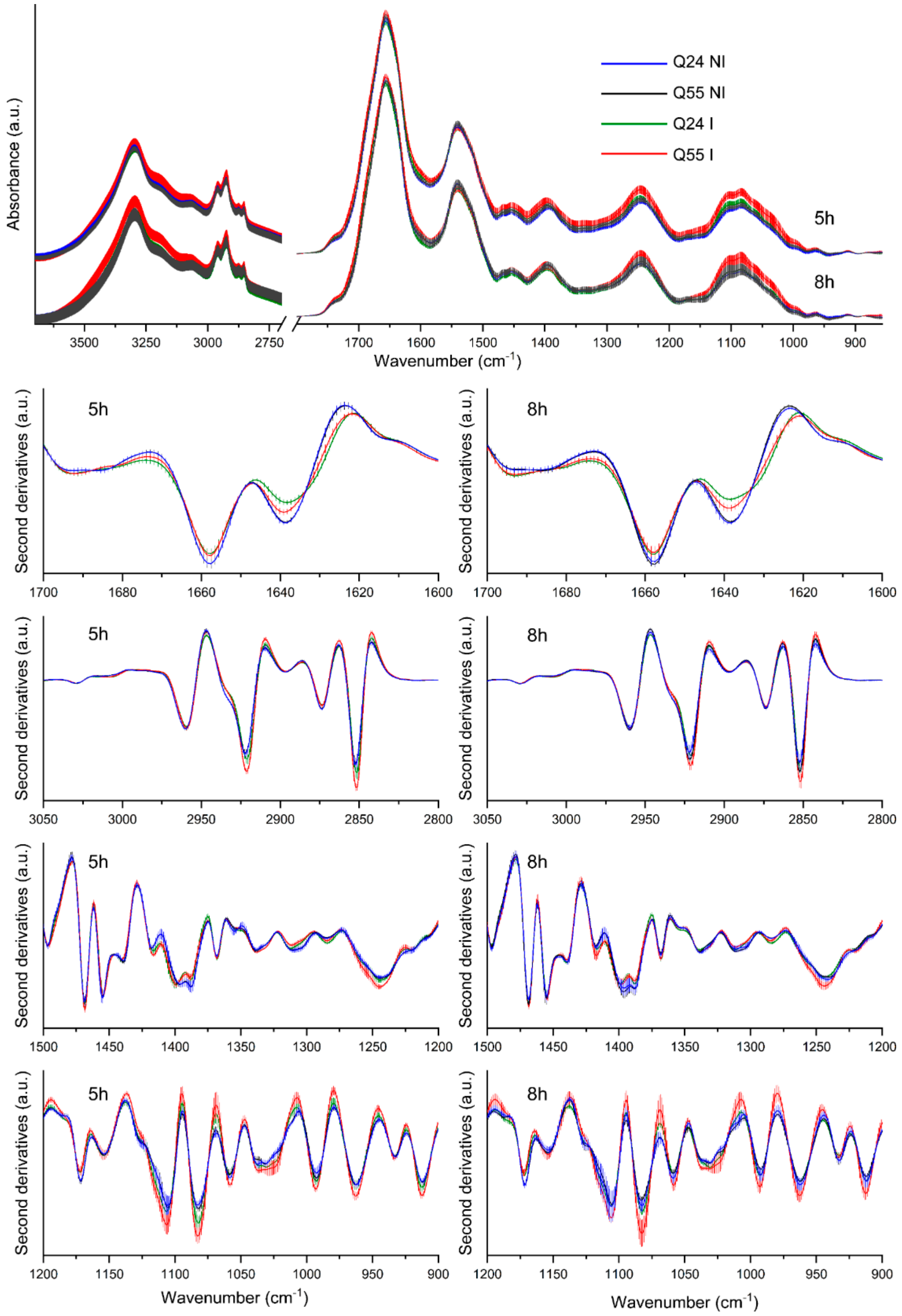

Figure S2: Absorption (top panel) and second derivative spectra (bottom panels) of *E. coli* cells expressing ATX3-Q24 and ATX3-Q55 at 5 and 8 hours of induction, and of NI cells. The average spectra and the standard deviation from three independent experiments are displayed.

Table S1: Peak position and assignment of the main spectral components identified by PLS-DA.

Wavenumber (cm^{-1}) from PLS-DA	Peak wavenumber (cm^{-1}) from second derivatives	Assignment	References
2854, 2852	2852	CH_2 symmetric stretching	[1]
1660-1654	1658	α-helices and random coils	[2]
1642-1640	1639	native β-sheets	[2]
1630	1630	native-like β-sheets and β-sheets in protein aggregates	[3]
1625		intermolecular β-sheets	[2,3]
1406, 1394, 1372–1378	1396, 1388	CH_2-CH_3 of lipid polar heads and hydrocarbon chains, as well as of peptidoglycans	[1,4,5]
1250	1243	PO2- antisymmetric stretching	[1,5]
1137, 1135, 1100	1105	Complex vibrational modes of carbohydrates in peptidoglycans	[4,5]
1070, 1058	1058	PO2- symmetric stretching and complex vibrational modes of carbohydrates in peptidoglycans	[1,4,5]
1044	shoulder of the band at 1039	Complex vibrational modes of carbohydrates in peptidoglycans.	[4,5]

1. Casal, H.L.; Mantsch, H.H. Polymorphic phase behaviour of phospholipid membranes studied by infrared spectroscopy. *Biochim Biophys Acta* **1984**, *779*, 381–401, doi:10.1016/0304-4157(84)90017-0.
2. Barth, A. Infrared spectroscopy of proteins. *Biochim Biophys Acta* **2007**, *1767*, 1073–1101, doi:10.1016/j.bbabio.2007.06.004.
3. Natalello, A.; Doglia, S.M. Insoluble protein assemblies characterized by fourier transform infrared spectroscopy. *Methods Mol Biol* **2015**, *1258*, 347–369, doi:10.1007/978-1-4939-2205-5_20.
4. Naumann, D.; Barnickel, G.; Bradaczek, H.; Labischinski, H.; Giesbrecht, P. Infrared Spectroscopy, a Tool for Probing Bacterial Peptidoglycan. *European Journal of Biochemistry* **1982**, *125*, 505–515, doi:https://doi.org/10.1111/j.1432-1033.1982.tb06711.x.
5. Kochan, K.; Perez-Guaita, D.; Pissang, J.; Jiang, J.-H.; Peleg, A.Y.; McNaughton, D.; Heraud, P.; Wood, B.R. In vivo atomic force microscopy-infrared spectroscopy of bacteria. *J R Soc Interface* **2018**, *15*, doi:10.1098/rsif.2018.0115.
